# Advanced Cellulose–Nanocarbon Composite Films for High-Performance Triboelectric and Piezoelectric Nanogenerators

**DOI:** 10.3390/nano13071206

**Published:** 2023-03-28

**Authors:** Jaime González, Ali Ghaffarinejad, Maxim Ivanov, Paula Ferreira, Paula M. Vilarinho, Ana Borrás, Harvey Amorín, Bernd Wicklein

**Affiliations:** 1 Materials Science Institute of Madrid (ICMM), Consejo Superior de Investigaciones Científicas (CSIC), 28049 Madrid, Spain; jaigon14@ucm.es (J.G.); hamorin@icmm.csic.es (H.A.); 2 Nanotechnology on Surfaces and Plasma Lab, Materials Science Institute of Seville (ICMS), Consejo Superior de Investigaciones Científicas (CSIC-US), 41092 Seville, Spain; 3 CICECO—Aveiro Institute of Materials, Department of Materials and Ceramic Engineering, University of Aveiro, 3810-193 Aveiro, Portugal; ivanovmaxim@ua.pt (M.I.); pcferreira@ua.pt (P.F.); paula.vilarinho@ua.pt (P.M.V.)

**Keywords:** cellulose, nanocarbon, nanocomposite, triboelectricity, piezoelectricity, nanogenerator, piezoresponse force microscopy

## Abstract

Natural polymers such as cellulose have interesting tribo- and piezoelectric properties for paper-based energy harvesters, but their low performance in providing sufficient output power is still an impediment to a wider deployment for IoT and other low-power applications. In this study, different types of celluloses were combined with nanosized carbon fillers to investigate their effect on the enhancement of the electrical properties in the final nanogenerator devices. Cellulose pulp (CP), microcrystalline cellulose (MCC) and cellulose nanofibers (CNFs) were blended with carbon black (CB), carbon nanotubes (CNTs) and graphene nanoplatelets (GNPs). The microstructure of the nanocomposite films was characterized by scanning electron and probe microscopies, and the electrical properties were measured macroscopically and at the local scale by piezoresponse force microscopy. The highest generated output voltage in triboelectric mode was obtained from MCC films with CNTs and CB, while the highest piezoelectric voltage was produced in CNF-CNT films. The obtained electrical responses were discussed in relation to the material properties. Analysis of the microscopic response shows that pulp has a higher local piezoelectric d_33_ coefficient (145 pC/N) than CNF (14 pC/N), while the macroscopic response is greatly influenced by the excitation mode and the effective orientation of the crystals relative to the mechanical stress. The increased electricity produced from cellulose nanocomposites may lead to more efficient and biodegradable nanogenerators.

## 1. Introduction

The rapid expansion of low-power electronic devices such as sensors, healthcare monitoring systems, actuators, wireless transmitters and IoT appliances amongst a plethora of further applications increases the demand for autonomous power sources beyond batteries [1]. Among others, cost-effective nanogenerators based on triboelectricity [2] and piezoelectricity [3] have come into focus for this purpose as they are capable of converting ambient mechanical motion into electrical energy with very high efficiency. Triboelectricity is produced when two materials come into contact and charges of opposite signs build up spontaneously on each material’s surface [2]. The origin of these charges is still debated, being attributed to electron, ion and/or material fragment transfer [4]. Upon separation, a potential difference arises, which initiates an electrostatically induced current through an external circuit. Piezoelectricity, on the other hand, appears under mechanical excitation that distorts the intrinsic dipole moment in a noncentrosymmetric crystal structure, giving rise to an electrical response (i.e., electrical charge or voltage) [3]. The resulting surface charge is balanced through a current in the external circuit, which can be used to power electronic devices or stored in batteries. Both types of nanogenerators rely mostly on synthetic polymers (PVDF, PTFE, FEP, PVC, PDMS, etc.) or inorganic solids (PZT, ZnO, BaTiO_3_, ZnSnO_3_, etc.) [3,5,6].

However, many of these conventional materials may represent environmental issues such as toxicity, resource depletion and non-recyclability. In recent years, paper-based electronics and devices have been developed that feature important characteristics such as renewability, abundance, biodegradability, wearability, mechanical flexibility and strength [7,8,9,10]. All these may finally result in a smaller material footprint of power supply devices and may eventually reduce the problem of electronic waste [11]. Specifically, cellulose and polysaccharide-based triboelectric nanogenerators (TENGs) [12,13,14] and piezoelectric nanogenerators (PENGs) [15,16] have received much attention as potential alternatives to conventional nanogenerators.

Cellulose may have both tribo- and piezoelectric properties [15]. In fact, cellulose and paper appear slightly off the neutral position on the positive side in the so-called triboelectric series [5,6]. These phenomenological observations suggest that cellulose tends to donate electrons to stronger withdrawing materials upon contact or friction. It is believed that oxygen atoms are responsible for ceding electrons and thus causing a net positive triboelectric potential in cellulose [15]. Hence, pairing cellulose with highly electronegative materials such as halide or PDMS-containing materials can render increased energy output [12,13,17].

On the other hand, piezoelectricity in wood cellulose was described as early as 1955 by Fukada [18]. This property originates from the noncentrosymmetric arrangement of polar hydroxyl groups in the cellulose I crystal [19]. A net dipole moment is created by three kinds of hydrogen bonding interactions between O2–H and O6, O3–H and O5 and O3–H and O6 [20] which lie in the *b*,*c* plane of the I_β_ monoclinic crystal cell of wood and plant-based cellulose [15,19]. This involves a hydrogen bonding plane in *x* and *y* directions along the longitudinal orientation of a cellulose crystal. Mechanical deformations relative to this plane cause a potential difference (voltage) parallel (i.e., longitudinal d_33_ piezo-coefficient) or normal (i.e., transverse d_31_ piezo-coefficient) to the direction of the mechanical excitation. 

Yet, the lower performance of cellulose in TENGs and PENGs versus synthetic polymers is still a critical obstacle for the wider deployment of cellulose [15]. For instance, cellulose was reported to have a d_33_ value of 0.4 pC/N as compared to 20–30 pC/N of PVDF [19]. In addition, TENGs using pure cellulose produce low peak voltage (5–20 V) and low power output (1–100 mW/m^2^) values [21,22], while more tribopositive polymers such as nylon, PVA, PMMA and PEO may render peak voltage of several hundred volts and power density in the range of 0.1–10 W/m^2^ [6]. One possible mitigation strategy to enhance these properties consists in the addition of dielectric or conductive fillers to the cellulose matrix. Dielectric fillers may increase the relative permittivity (ε_r_) and density of intrinsic oriented dipoles [23,24], while conductive fillers may increase ε_r_, the charge density (σ_o_), electron mobility (μ_e_), volume and surface conductivity and induce the nucleation of ferroelectric phases (e.g., ß-PVDF) [25,26,27] of the respective nanocomposites. For instance, nanocarbon materials are highly conductive, offer mechanical reinforcement and show vast morphological variety [28,29,30]. Following this strategy, carbon black has been combined with different cellulose derivatives to produce functional nanocomposites [31,32]. On the other hand, promising TENGs based on low-dimensional nanocarbon fillers have been reported [33], but so far there are only limited studies on TENGs and PENGs based on cellulose–nanocarbon composites. For instance, a nanocomposite of bacterial cellulose/BaTiO_3_/CNTs or of carboxymethylated cellulose/PDMS/CNTs was tested in a PENG [34,35]. However, in general, paper or cellulose was either used as a passive substrate for active materials (PET, PTFE, PVDF, Cu, etc.) or as an active component using plain cellulose films [12,14,15]. Therefore, a systematic investigation that studies cellulose–nanocarbon composites for these applications is still required. Specifically, the research question of how the different parameters such as morphology, size and dimensionality of the components may influence the electrical output needs to be given more attention.

In this article, we will approach such a challenge by combining three types of celluloses and three types of nanocarbon fillers to prepare nine different cellulose–nanocarbon composite films. The celluloses are eucalypt wood pulp fibers (cellulose pulp (CP)), microcrystalline cellulose (MCC) and cellulose nanofibers (CNFs) that exhibit fiber diameters from tens of microns down to a few nanometers [36]. The nanocarbon fillers are graphene nanoplatelets (GNPs), carbon nanotubes (CNTs) and carbon black (CB) with 2D to 0D morphology, micron- to nanoscale diameters and different electrical conductivities [28,29,30]. With these composites, both TENG and PENG devices were built and their performance for conversion of low-frequency vibrations and deformations (i.e., below 20 Hz) was analyzed. This study aims at discussing this performance in relation to the micro- and macroscopic film properties. The significance of this study resides in exposing the most important factors that influence the electrical response of cellulose–nanocarbon composites. The results suggest that factors such as the specific surface area of the filler, nanoscale dispersion, filler content, onset of conductivity and matrix porosity have a stronger influence on the triboelectric output voltage than only the electrical conductivity of the fillers. Cellulose crystallinity and crystal orientation are additional important factors that influence the PENG performance.

## 2. Materials and Methods

### 2.1. Materials

Bleached commercial Eucalyptus globulus kraft pulp was obtained from La Montañanesa (Grupos Torraspapel, Zaragoza, Spain). Microcrystalline cellulose (MCC, ref. 435236-250G) and cetyltrimethylammonium bromide (CTAB) were purchased from Sigma Aldrich (Madrid, Spain). Multiwalled carbon nanotubes (DRP-MWCNT, 95% purity) were supplied by Metrohm Dropsens (Oviedo, Spain). Graphene nanoplatelets (KNG-150, 98% purity) were acquired from Knano (Xiamen, China). Carbon black (Conductex 975) was purchased from Columbian Chemicals Company (Marietta, GA, USA).

### 2.2. Material Preparation

**Figure 1:**
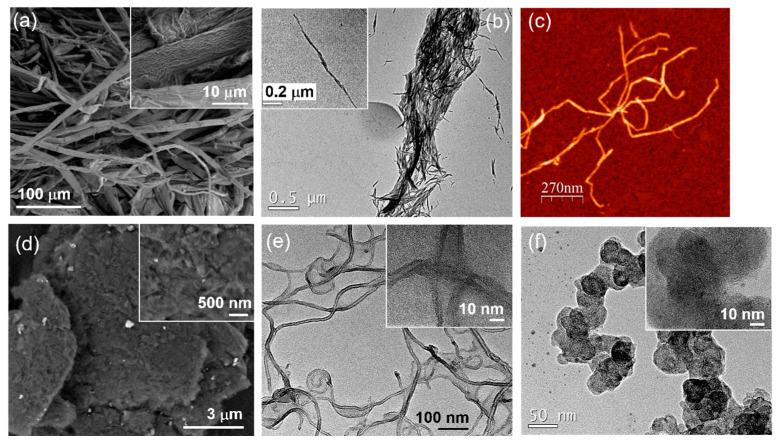
Morphology of starting nanomaterials. SEM image of cellulose pulp and high magnification of the fiber surface (inset) (**a**), TEM image of microcrystalline cellulose (**b**), AFM image of cellulose nanofibers (**c**), SEM image of graphene nanoplatelets (**d**), TEM images of multiwalled carbon nanotubes (**e**) and carbon black (**f**). High-magnification images are inset. The nanocarbon materials were sonicated and stabilized with 0.4 mg/mL CTAB prior to observation.

The cellulose pulp (CP) was dispersed in water at 3 mg/mL under mechanical stirring for 48 h. Cellulose nanofibers (CNFs) were prepared as reported previously [36] from bleached eucalypt pulp resulting in aqueous dispersions containing CNF with a charge density of 900 mequiv/g. The CNF dispersion was diluted to 3 mg/mL with help of an UltraTurrax before mixing with the nanocarbon fillers. Aqueous MCC dispersions of 2 wt% were prepared with an ultrasound probe (Vibra Cell, Sonics, Newtown, CT, USA) for 15 min or 20 kJ energy input and then diluted to 3 mg/mL. Note that in this study all three celluloses were dispersed, maintaining their fibrous morphology (see Figure 1a–c), and did not dissolve into a polymer solution. The nanocarbons were dispersed at 0.2 mg/mL in water containing 0.4 mg/mL CTAB and sonicated.

### 2.3. Film Formation

Appropriate volumes of the dispersions of nanocarbon fillers (GNPs, CNTs, CB, all at 0.2 mg/mL) were added to 10 mL of dispersions of the cellulose materials (CP, MCC, CNF, all at 3 mg/mL) to render composite mixtures with different cellulose/nanocarbon ratios of 0–0.05 (*w*/*w*). The CP- and MCC-based composite mixtures were probe-sonicated (6 kJ and 8 kJ, respectively), while the CNF-based composite mixtures were homogenized in a Thinky Mixer ARA-250 (Japan) for 5 min at 2500 rpm. Then, the CP and MCC mixtures were vacuum-filtered through a Sefar-Nitex 03/10–2 microfiber fabric (Sefar AG, Switzerland), while the CNF mixtures were pressure-filtered through a cellulose ester membrane (Millipore RAWP, 0.45 μm pore size). In the case of the CNF-based films, a solvent exchange was performed (water to isopropanol) with the wet filter cakes to reduce the surface defects during film drying. All films were oven-dried for 24 h at 40 °C.

### 2.4. Characterization

The apparent density, ρ_a_, of the films was calculated from the external volume (thickness and area measured with a digital caliper) and the mass of the samples. The porosity was calculated as (1 − ρ_a_/ρ_s_) with ρ_s_ as the skeletal density of the nanocomposite films. The ρ_s_ was estimated as the weighted ratio of the true densities of cellulose (1.4 g/cm^3^ [37]). and the carbon allotropes (1.75–2.25 g/cm^3^, see Table S1). Raman spectroscopy was performed with a mSense-LabC1X Enwave Optronics Raman confocal microscope (BX 51M, Olympus) equipped with a ProRaman-L (ChemLogix) laser generator (532 nm). Electron microscopy was performed with a field-emission scanning electron microscope (SEM) from FEI (NOVA Nano SEM 230) and a transmission electron microscope (JEOL JEM 2100). Local topography and piezoelectric properties were studied using the SPM technique. Topography images of individualized CNFs were obtained in the dynamic mode of atomic force microscopy (AFM, Cervantes, Nanotec Electrónica S.L. equipped with a PPP-FMR (force constant of 1.5 N/m, resonance constant of 70 kHz)). Piezoresponse force microscopy (PFM) measurements on freestanding films were performed in contact AFM (Ntegra NTMDT). The ElectriMulti75-G (Budget Sensors) probes (resonant frequency of 75 kHz, force constant of 3 N/m) coated with Cr/Pt were utilized. The DC bias voltage applied to the sample during the measurements varied in the range of 1–10 V. The PFM measurements were performed with an AC voltage frequency of 50 kHz and an amplitude of 10 V. All the SPM measurements were performed under controlled environmental conditions (30 °C, humidity ~40%). The film thickness was measured with a high-precision caliber (Mitutoyo) and averaged over five independent measurements in different areas of the films.

### 2.5. Electrical Measurements

The electrical conductivity of the nanocomposite films was measured by a 4-probe setup connected to a Solartron 1480 potentiostat (MultiStat). The triboelectric characteristics of the cellulose–nanocarbon films were studied in a vertical contact–separation mode with a mixed cellulose ester (MCE) membrane (VSWP, Millipore) as a counterpart. The 3 × 3 cm^2^ films were glued with conductive double-sided tape (9711S, 3M) on Cu tape as a current collector and mounted on acrylate supports. The MCE film was attached to the vertically moving shaft of an electromagnetic shaker (LDS-201V, Brüel & Kjær) and periodically contacted with the nanocomposite film. The shaker was controlled by a function generator (RSDG805, RS Pro) working at a controlled frequency (sine wave, 10 Hz). The force (10 N) was measured by an IEPE transducer (DYTRAN 1053V2) and registered with an oscilloscope (TBS-1052C, Tektronix). The output voltage was measured with the same oscilloscope through a 10 MΩ probe. The output current was determined by registering the voltage drop across an adjustable resistor (decade resistance box RM6-N3, Cropico) with an oscilloscope through a 100 MΩ probe and using Ohm´s law. The mean power, *P_av_*, was calculated as

$$P_{av}\left(t\right)=\int_{T}^{2T}\frac{V_{L}^{2}\left(t\right)/R_{L}}{T},$$

where *V_L_*(*t*) is the voltage across the resistive load *R_L_* and *T* is the period of actuation. The piezoelectric output of the cellulose–nanocarbon films was investigated with the same shaker setup only that the films were sandwiched between the conductive adhesive tape (9711S, 3M), the Cu current collectors and the acrylate supports. The piston of the vertically moving shaker tapped at a controlled frequency and force on the samples, and the output voltage, current and *P_av_* were measured as described above. The tapping frequency and force were 10 Hz and 10 N if not stated otherwise. In addition, the piezoelectric response of some films was also determined in a cantilever setup. The films were electrically contacted on both sides with conductive adhesive tape (9711S, 3M), packaged in flexible plastic foil and attached to a rectangular steel sheet as a cantilever beam. The cantilever was attached to the shaker (Smart Shaker K2007E01 from The Modal Shop, USA) and operated at the resonance frequency of 17 Hz of the beam. The generated voltage and current were registered with a Tektronix TDS1052B oscilloscope and Keithley 2635A source meter.

## 3. Results and Discussion

### 3.1. Morphology of the Starting Materials

The morphology of the starting materials was characterized by electron and atomic force microscopy. All three celluloses (CP, MCC and CNF) are fibrous, albeit at different length scales. Cellulose pulp (CP) consists of flat fibers with a width of about 10 μm and a corrugated surface (Figure 1a), while microcrystalline cellulose (MCC) consists of needle-like particles that are about 30 nm thick and 200–400 nm long (Figure 1b). Cellulose nanofibers (CNFs) are several hundred nanometers long, are 2–3 nm in diameter and show kinks and bends along the fiber which give flexibility to CNF (Figure 1c). The three nanocarbons show differences in both morphology and dimensionality. Graphene nanoplatelets (GNPs) are two-dimensional (2D) flakes of about 5–20 μm diameter, which consist of stacked and compacted graphene sheets (Figure 1d). These platelets have a thickness of tens to hundreds of nanometers, suggesting a low degree of exfoliation [28]. Multiwalled carbon nanotubes (CNTs) are 7–12 nm in diameter and 1–2 μm long (Figure 1e), whereas carbon black (CB) consists of 30–40 nm sized particles that tend to agglomerate (Figure 1f). High-resolution TEM reveals the layered graphite structure of the CNF walls (inset Figure 1e) and the crumpled carbon layers of carbon black (inset Figure 1f). Raman spectra show the typical bands for all these nanocarbons (Figure S1).

### 3.2. Nanocarbon Dispersions

**Figure 2:**
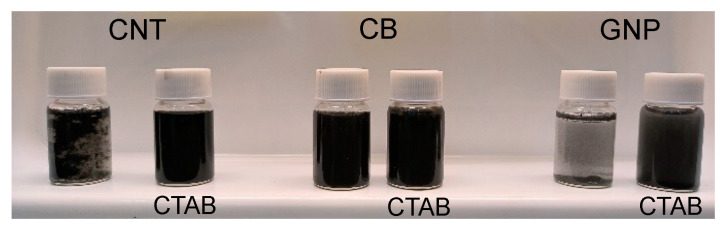
Probe-sonicated nanocarbon dispersions (0.2 mg/mL) without (left vials) and with (right vials) 0.4 mg/mL CTAB.

In order to obtain nanocomposites with individualized, non-aggregated and non-segregated components, both the celluloses and the nanocarbon fillers need to be dispersed individually as colloidally stable suspensions before the suspensions are mixed in the appropriate ratios. The celluloses are readily dispersed in water by a combination of stirring, high-shear homogenization and probe sonication. On the other hand, the colloidal stability of the nanocarbon dispersions was ensured by a further addition of 0.4 mg/mL CTAB in combination with probe sonication. Figure 2 shows the nanocarbon dispersions without and with CTAB. The latter results in stable dispersions, while the former leads to flocculation and particle sedimentation.

### 3.3. Microstructure of the Nanocomposite Films

**Figure 3:**
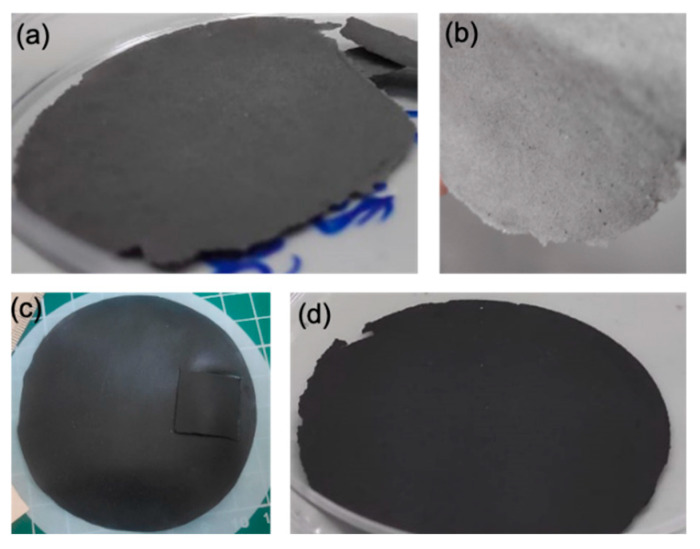
Digital photographs of nanocomposite films. Images of MCC-1%CB (**a**), CP-0.05%CNT (**b**), CNF-1%CB (**c**) and MCC-5%CNT (**d**).

**Figure 4:**
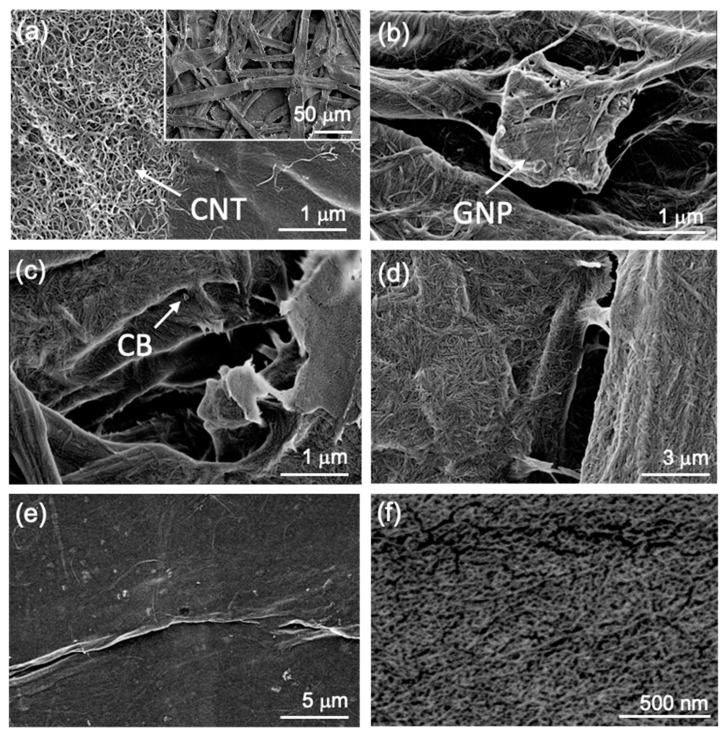
Microstructure of the nanocomposite films. SEM images of CP-0.2%CNT (inset at low magnification) (**a**), CP-0.3%GNP (**b**), MCC-0.1%CB (**c**), MCC-0.2%CNT (**d**), CNF-0.3%CB (**e**) and CNF-0.4%CNT (**f**).

The nanocomposite films were prepared by filtration of nine combinations of the three types of celluloses and the three different nanocarbon fillers. Examples of the resultant nanocomposite films are shown in Figure 3. In all cases, the uniform grey-to-black color suggests a homogeneous distribution of the carbon fillers in the cellulose matrices at the macroscopic scale. This can be attributed to the stabilized colloidal suspensions of the nanocarbons that are compatible with the aqueous cellulose dispersions. The textural properties of representative cellulose–nanocarbon films are summarized in Table 1. Most notably, the porosity decreases from CP- (52%) and MCC- (35%) to CNF-based composite films (5%). Accordingly, the thicknesses of the various composite film types are 72 μm (CP), 40 μm (MCC) and 24 μm (CNF), and the apparent densities are 0.69, 0.95 and 1.39 g/cm^3^, respectively. The CNF films are the most compact, which can be attributed to the nanosize and flexibility of the fibers along with the strong interfibrillar hydrogen bonding [38]. CP consists of micron-sized fibers, which do not compact as densely as CNFs and lead to more pores (see also Figure 4a). MCC, on the other hand, tends to aggregate due to a lack of ionizable surface groups, and the stiff needle-like particles further reduce the packing density, hence resulting in more porous films. Note that the fabrication of these composite films could also be scalable both in size and number through common paper-making processes [39], which is an important aspect for commercial paper-based energy applications.

The microstructure of the nanocomposite films was characterized by SEM microscopy of the film surfaces. Figure 4 displays exemplary films from combinations of three celluloses with different nanocarbon fillers. The CP films reveal an uneven surface with micron-sized pores and gaps between the pulp fibers. Closer inspection shows the deposition of CNTs on the surface of individual pulp fibers (Figure 4a), whereas GNPs are partially covered by fine pulp fibers (Figure 4b). These so-called fines are sub-micron thin pulp fibers that are produced during mechanical pretreatments of industrial cellulose pulps such as the one used in this study [36]. The MCC films consist of compacted cellulose crystals with dimensions in the nanoscale in agreement with the TEM observations (cf. Figure 1b). These small dimensions make the observation of the embedded nanocarbons difficult. For instance, Figure 4c shows some CB agglomerates, while the CNTs could not be distinguished from the MCC matrix (Figure 4d). As in the pulp films, there are also pores visible. On the contrary, the CNF films display a smooth and non-porous surface, which is typical for such films. The 3 nm thick fibers effectively assemble into dense films owing to their nanoscale flexibility and attractive H-bonding of surface groups (OH, COONa) and water bridges [38]. As in the MCC films, the nanocarbon fillers could not be distinguished from the CNF matrix (cf. Figure 4e,f).

### 3.4. Electrical Conductivity

The electrical conductivity of the nanocomposite films as a function of the nanocarbon content was determined with the four-probe method (Table 2). With increasing nanocarbon filler content, the conductivity of the nanocomposite films increases, as exemplified for MCC-CNT (Figure S2). The onset of electrical conductivity is 20 μS/cm at 0.4 wt% CNTs and rises to 4.9 S/cm at 5 wt% CNTs. These values are in good agreement with those reported previously for cellulose–nanocarbon composites [40,41,42,43]. In general, the electrical percolation threshold (EPT) of CNTs in CP and MCC is lower than that of CB (0.3–0.4 vs. 1 wt%). A similar observation has also been made by Imai et al. [42] for CNTs vs. CB in cellulose pulp films. This phenomenon can be attributed to the higher intrinsic conductivity of CNTs as compared with CB (Table S1) and to the fact that 1D fillers percolate at a lower concentration than spherical fillers [44,45]. In the case of GNPs, a concentration up to 0.5–1 wt% did not induce electrical conductivity in the nanocomposite films regardless of the cellulose type. The low degree of exfoliation of these GNPs results in isolated flakes (cf. Figure 4b), which do not percolate in this concentration range. In addition, 2D materials such as GNPs have a 2-fold higher theoretical EPT than 1D fillers [46] requiring concentrations well above 2%, as reported for GNP-CNF [47] and GNP-lyocell [48].

On the other hand, it can be observed that the onset of percolation is also in part influenced by the cellulose matrix. For instance, in CP and MCC films, the EPT is significantly lower than that in CNF films. The percolation threshold of CNTs is 0.3–0.4 wt% and 1 wt% of CB in CP and MCC, respectively, while 2.5 wt% of CNTs is needed in CNF to attain 0.25 S/cm. No conductivity was measured in CNF with 1 wt% CB or GNPs. This observation points to the significant influence of fiber morphology and film compaction on the EPT. In dense films consisting of nanosized fibers, a higher amount of the conducting filler is needed to form an electrical conduction path. For instance, it was reported that a content of 6 wt% MWCNTs in CNF was needed for percolation (10^−5^ S/cm) [49], while elsewhere it was 3 wt% MWCNTs (10 S/cm) [50]. In these films, the nanocarbon fillers are more dispersed, and the electrical conduction paths are intersected by the nonconducting cellulose nanofibers. On the other hand, in the CP and MCC films, the fibers are larger and more aggregated. This confines the fillers to fewer but thicker conduction paths at the same filler content. Similar effects have been observed for electrical percolation in fine- and coarse-grained ceramics [51,52].

### 3.5. Triboelectric Properties

**Figure 5:**
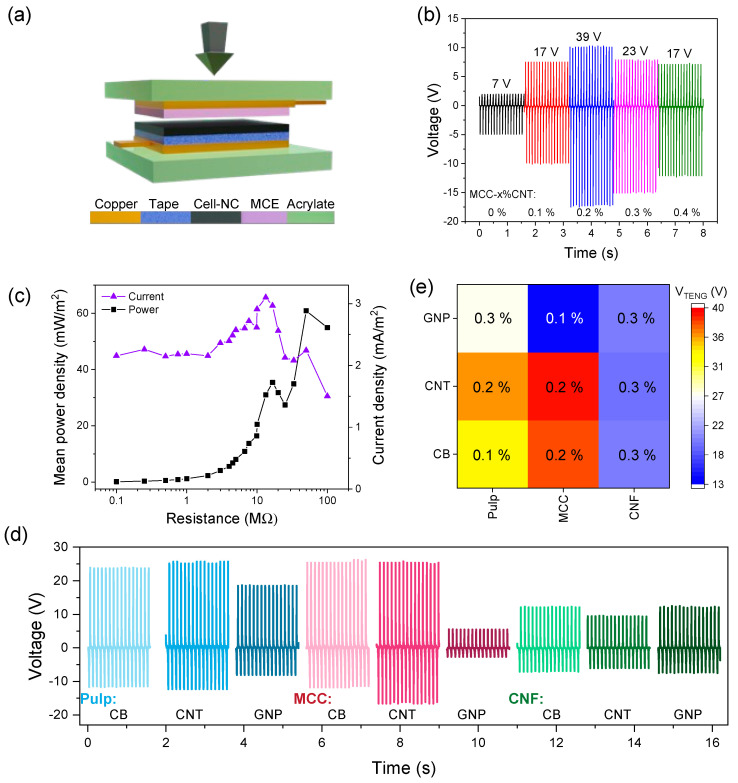
Triboelectric harvesting measurements. Illustration of the contact–separation mode TENG with MCE and the cellulose–nanocarbon (Cell-NC) nanocomposite films as triboactive components (**a**). Triboelectric output voltage of MCC as a function of the CNT filler content (**b**). Power and current density curves of MCC-0.2%CNT (**c**). Output voltage of all cellulose–nanocarbon combinations (**d**). Heat map of the maximum triboelectric output voltage at the optimum nanocarbon content of each cellulose–nanocarbon combination (**e**).

The triboelectric voltage of the nanocomposites was determined in the vertical contact–separation mode [53], where a mixed cellulose ester (MCE) film is attached to the moving piston of an electromechanical vibrator and touches the cellulose nanocomposite films under controlled force (10 N) and frequency (10 Hz) (Figure 5a). Measurements with an electrostatic voltmeter show that the electrostatic surface potential of the MCE film is highly negative (−2 kV), whereas cellulose is slightly positive, in agreement with many triboelectric series [5,6]. This suggests that upon contact, triboelectrification forms charges of contrary signs at the surfaces and the system is in electrical equilibrium. As the two surfaces are separated, the transferred charges on the top surfaces of both materials induce charges on their respective bottom electrodes by electrostatic induction, which causes an electron flow in the external circuit attached to the electrodes [2].

#### 3.5.1. Performance Measurements

Measurements show the corresponding voltage oscillations during the contact and separation process (Figure 5b,d). It should be remarked that potential piezoelectric contributions to the triboelectric voltage signal, corresponding to mode 1 of hybrid nanogenerators [54], are likely to be minimal given their smaller output range (see Section 3.6). The addition of the nanocarbon fillers clearly enhances the triboelectric voltage of the cellulose films, as demonstrated for MCC-CNT films (Figure 5b). The TENG output voltage rises from 7 V for pure MCC to 39 V for MCC-0.2%CNT, which is an increase of 450%. At higher CNT content, the voltage decreases again. The current and mean power densities of MCC-0.2CNT are shown in Figure 5c. The maximum current produced by the film is 3 mA/m^2^, and the mean power density is 60 mW/m^2^ at 40 MΩ. Figure 5d shows the maximum triboelectric output voltage curves for each of the nine combinations. Figure 5e depicts a heatmap of the maximum voltage at the optimum filler content of each cellulose–nanocarbon combination (the heatmap is constructed from the comprehensive voltage vs. filler content data in Figure S3). The heatmap allows for rapid identification of the voltage output pattern across the various nanocomposites. The highest values are obtained for MCC films (38 V), then CP (36 V) and lastly CNF (20 V) films.

The output performance is compared with published cellulose-based TENGs. In general, pure cellulose (i.e., CNF, MCC, bacterial cellulose (BC), paper) was reported to deliver 5–20 V, while blending with PDMS, PVA or other polymers may render up to 600 V. The addition of ferroelectric fillers such as BaTiO_3_ can produce up to 90 V, and 2D fillers such as phosphorene render 2 V [14,15,22,55]. The output power in these cases was 5 mW/m^2^ for BC [14], 140 mW/m^2^ for CNF [22] and up to 94 mW/m^2^ for nanocellulose/phosphorene composites [55]. These values show that the performance of MCC-CNT surpasses that of pure celluloses and is in the range of other cellulose–nanofiller composites.

#### 3.5.2. Film Properties and Performance

The triboelectrical responses are discussed in relation to the film properties such as microstructure and porosity, filler content, electrical conductivity and filler properties. The observation of a maximum output voltage (Figure 5b) is commonly reported for nanocomposites with increasing filler content, be it dielectric [25,26] or conducting (CNT) [23,24] fillers. In agreement with the lumped model of TENGs [56,57], increased relative permittivity and charge density are claimed as reasons for the rise. For instance, the addition of nanocarbon fillers below the EPT concentration was reported to enhance ε_r_, σ_o_ and μ_e_ of dielectric polymers [23,24,33]. On the other hand, particle agglomeration that may lead to conductive networks and accumulation on the film surface, which reduces the effective friction area of the dielectric matrix with the counter layer, is considered responsible for the subsequent decay [24]. Indeed, the maximum triboelectric voltage of MCC-CNT coincides with the onset of the electrical conductivity of the film (cf. Figure S2).

Another observation is that the type of filler and cellulose matrix influences the output voltage. For instance, CB and CNTs produce higher voltage than GNPs, whereas pulp- and MCC-based films render higher voltage than CNF-based films (38 V vs. 20 V). The higher voltage from CP and MCC films might be explained by their higher porosity. It has been reported that porous films show higher voltage as compared to their dense counterparts, which has been attributed to a higher relative permittivity and higher surface area, on which more triboelectric charge can be stored during each contact–separation cycle [58]. In addition, higher (open) porosity also increases the surface roughness of these composite films, as observed in the SEM images (Figure 4). Surface roughness and surface nanostructures are well known to enhance the triboelectric output of polymer films [2] and are likely to contribute also in this study.

The lower voltage output from GNP-containing films might be explained by their particle size and specific surface area (SSA). Nanocarbons have been reported to act as charge traps and micro-capacitors within polymer films, increasing the triboelectricity [33,57]. These effects are more pronounced in the case of nanoscopic materials that have a large SSA. This is the case for CB and CNTs (250 and 300 m^2^/g, respectively), while GNPs have only 80 m^2^/g due to their micrometric size (albeit the thickness is tens to hundreds of nanometers) (Table S1).

Concerning the electrical conductivity of nanocarbon fillers, the results show that the maximum output voltages are commonly obtained for compositions below the EPT. In the absence of a conducting network within the films, the starkly different conductivities of, for instance, CB (~10^−4^ S/cm) and CNTs (~10^1^–10^3^ S/cm, see Table S1), do not seem to influence the output voltage of the respective films much; i.e., the generated triboelectric voltage is similar. As for the CNF films, the filler type does not appear to be crucial, which might be attributed to the dense film surface [58]. This suggests that factors such as the filler SSA, nanoscale dispersion, filler content, EPT and matrix porosity have a stronger influence on the output voltage than only the electrical conductivity of the fillers.

To summarize the performance–property relationships, the output improvement occurs at a filler content below the onset of electrical conductivity, the nanosized fillers CNTs and CB are more effective than GNPs, and the porosity of the CP- and MCC-based composite films could explain their generally higher triboelectric output performance.

### 3.6. Piezoelectric Properties

**Figure 6:**
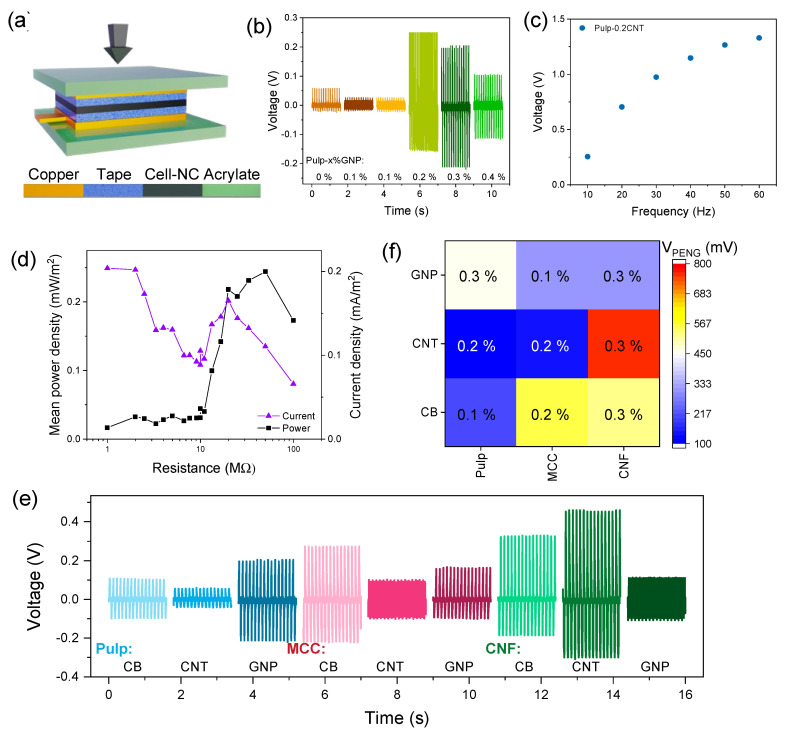
Piezoelectric harvesting measurements. Illustration of a PENG in tapping mode with the nanocomposite film between current collectors (**a**). Piezoelectric output voltage of pulp as a function of the GNP filler content (**b**). Voltage as a function of the tapping frequency of pulp-0.2%CNT (**c**). Current density and mean power density curves of pulp-0.3%GNP (**d**). Piezoelectric output voltage of all cellulose–nanocarbon combinations (**e**). Heat map of the maximum piezoelectric output voltage at the optimum nanocarbon content of each cellulose–nanocarbon combination (**f**).

The macroscopic piezoelectric behavior of the nanocomposite films was studied in tapping mode, where the film is sandwiched between electrodes and mechanically compressed at a controlled frequency (10 Hz) and force (10 N) (Figure 6a). The piezoelectric response is measured parallel to the mechanical excitation direction, which corresponds to the longitudinal piezoelectric coefficient d_33_ [15].

#### 3.6.1. Performance Measurements

The results show that small addition of nanocarbon fillers generally increased the piezoresponse of the different cellulose films (Figure S3). For instance, 0.2 wt% of GNP content in CP films enhances the output voltage by a factor of 6 (Figure 6b). Increasing the GNP content beyond 0.3 wt% again reduces the voltage output. While pulp films were not conducting on the macroscopic level until 0.5 wt% GNPs (cf. Table 1), local short-circuiting of conductive pathways across the film could be responsible for leakage currents [27] and concomitant output voltage decay at >0.3 wt% GNPs. The tapping frequency was shown to influence the piezoresponse, as exemplified for pulp-0.2%CNT. Increasing the tapping frequency results in higher voltage values (Figure 6c). This phenomenon is commonly observed in piezo- and triboelectric nanogenerators and is attributed to increased charge transfer rates at higher frequencies [2,17]. The current and power–load curves of pulp-0.3%GNP are shown in Figure 6d with maxima of 0.2 mA/m^2^ and 0.25 mW/m^2^ at 50 MΩ. The piezoelectric output voltage curves of all nine combinations are presented in Figure 6e together with a piezoelectric voltage heatmap (Figure 6f), where the maximum voltage at optimized nanofiller content is represented for each nanocomposite. It can be noted that the nanocarbon content is between 0.1 and 0.3 wt% and the highest voltage up to 0.8 V is obtained with CP-GNP, MCC-CB and CNF-CNT. It appears that each type of cellulose works best with a specific nanocarbon filler and that all fillers are capable of enhancing the intrinsic, albeit small, piezoelectricity of cellulose. 

The performance of the nanocomposites is compared with cellulose-based PENGs in the literature. For instance, regenerated cellulose, BC and CNF produced 2, 14 and 15 V, respectively [16,34,59], while the addition of MoS_2_ nanosheets to CNF increased the PENG output to 42 V [16]. The output power density of a BC/BaTiO_3_/CNT nanocomposite was 12 mW/m^2^ and 13 mW/m^2^ for CNF/MoS_2_, while carboxymethylated cellulose compounded with PDMS and CNTs delivered 30 V and 18 μW/m^2^ [16,34,35]. These values are higher than those obtained in the present study, but it should be remarked that in these cases the main piezoelectric active materials were not cellulose but the additives (i.e., BaTiO_3_, MoS_2_, PDMS), which may explain the difference.

#### 3.6.2. Mechanism of Piezoelectricity in Cellulose Nanocomposites

The origin of the observed piezoelectricity and the mechanism of its enhancement in the cellulose nanocomposites might be rationalized by the combination of the intrinsic dipole moment of the cellulose crystals and the film processing. Vacuum filtration leads to a conformation of the cellulose fibers and crystals within the plane of the resultant film, where a fraction of the crystals are oriented with their dipole moment normal to the surface. Mechanical tapping on the films induces a change in these dipoles, and consequently, a relatively small piezoelectric surface charge originates at the film surface, which is compensated by opposite charges from the external circuit.

Adding small amounts of conductive fillers such as nanocarbons has been shown to enhance the piezoelectric output performance in some piezoelectric polymers and inorganic solids [27,60]. One of the suggested mechanisms is the formation of non-percolating conductive paths within the matrix that facilitates the charge transfer from the internal dipoles to the material surface and the efficient electrical contact with the external electrodes [27]. The issue of the optimized surface conductivity and material/electrode contact is especially relevant for porous films such as electrospun fiber mats [60] and paper-like materials such as the ones investigated here. Indeed, the results in Figure 6f seem to corroborate the surface conductivity effect. The maximum piezoelectric output is obtained at filler concentrations well below the percolation threshold. For instance, for CP-CNT, the EPT is 0.3% CNT, while the maximum output is achieved at 0.1% CNTs.

#### 3.6.3. Film Microstructure and Performance

Microstructural properties such as cellulose crystallinity, porosity and crystal orientation (see Section 3.6.4) and their possible influence on the piezoelectric response are discussed in the following. It appears that CNF nanocomposite films tend to generate slightly higher voltage than the other celluloses, with CP composite films having the lowest values (Figure 6e). Possible reasons might be related to the different crystallinity of the pulp and the cellulose nanofibers, the higher density of the CNF films and the effective orientation of the cellulose crystals (i.e., their dipole moment) relative to the mechanical excitation direction. The piezoelectricity of cellulose arises from its noncentrosymmetric crystal structure [15] and increases with crystallinity. As shown by Fillat et al. [36], the crystallinity of eucalypt CNF is lower than that of the eucalypt pulp, 57% vs. 81%, due to the hypochlorite treatment during the nanofibrillation process. Hence, it could be expected that pulp shows higher piezoelectricity than CNF films, but the contrary is observed.

The different porosity of the dense CNF and the porous pulp films could be another explanation. However, it was reported that porosity in both inorganic and polymeric films generally increases the piezoelectric response [61,62,63], including in cellulose aerogels [16]. It was reasoned that the internal voids enhance the strain on the crystal structure and thereby increase the piezoelectricity. Again, an opposing behavior is observed in the present study.

#### 3.6.4. Cellulose Orientation and Piezoresponse

**Figure 7:**
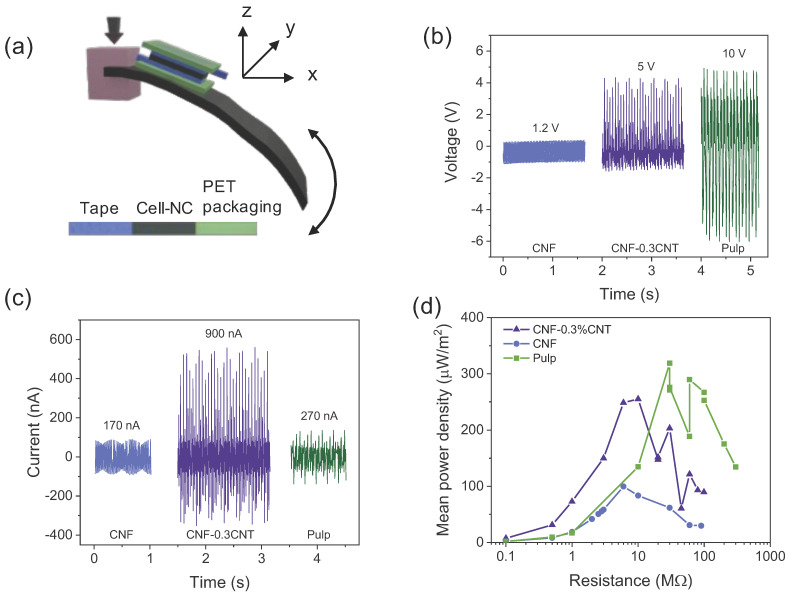
Cantilever excitation of piezoelectric response. Illustration of cantilever setup with sandwiched film between current collectors (**a**). Piezoelectric open-circuit voltage (**b**), short-circuit current (**c**) and power–load curves (**d**) obtained from pulp, CNF and CNF-0.3%CNT films.

Concerning a possible effect of the cellulose crystal orientation on the piezoresponse, CNF and CP films were investigated in a cantilever setup. In this excitation mode, the films were strained and compressed in the *x* direction and the piezoelectric response was measured in the *z* direction (Figure 7a). In this study, the transverse piezoelectric coefficient d_31_ is supposed to give the main contribution to the generated signal, but other piezoelectric tensor components were also possibly activated through sample bending [3]. The open-circuit voltage curves in Figure 7b show that CNF has the lowest voltage (1.2 V), which increases to 5 V with the addition of 0.3% CNTs, and that CP delivers 10 V. It can be noted that the values are significantly higher than those in the tapping mode, which are around 0.1–0.8 V. The current, on the other hand, is highest for CNF-0.3%CNT (Figure 7c), while the mean power density is highest for both CP (0.31 mW/m^2^) and CNF-0.3%CNT (0.26 mW/m^2^) and significantly lower for pure CNF (0.10 mW/m^2^) (Figure 7d).

Hence, a clear orientation dependence of the piezoresponse can be noticed; while in tapping mode (mainly probing of d_33_), CNF composites show a higher response than CP, in the cantilever mode (mainly probing of d_31_), the situation is the opposite. The cantilever mode is also much more effective in generating a piezoresponse (higher output voltage). In the tapping mode, only those crystals whose dipole moments lie parallel to the mechanical excitation contribute to the response. Contrarily, in the cantilever mode, the strain induces a response in all crystals parallel to the *x* direction (along the cantilever).

So far, it can be summarized that all types of nanofillers at a content below the EPT may promote a high response in a given cellulose matrix, that dense composite films produce higher responses than porous films and that cellulose crystallinity alone does not explain the macroscopic piezoresponses. Moreover, due to the crystal orientation dependence of the piezoresponse, the cantilever excitation mode is more effective in generating a high output voltage.

### 3.7. Piezoelectric Force Microscopy Measurements

**Figure 8:**
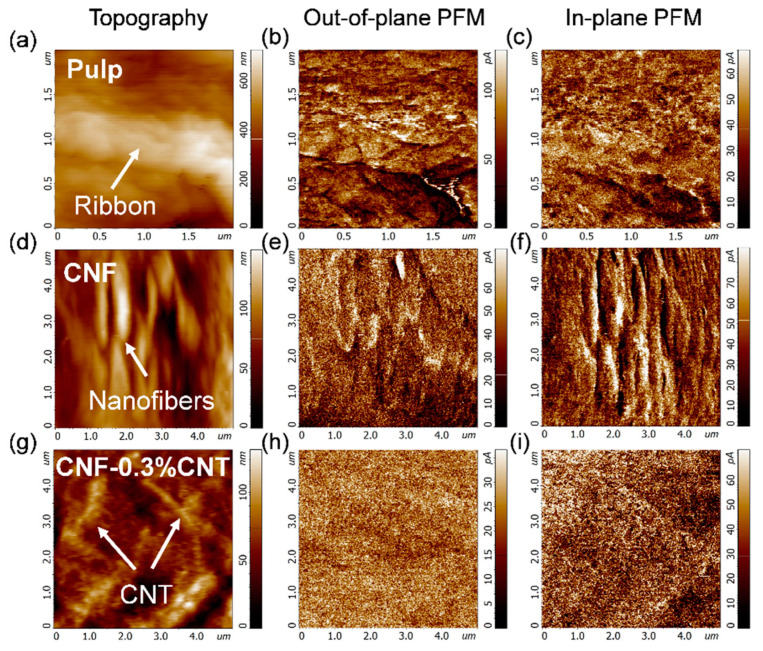
AFM images of the topography (**a**,**d**,**g**) as well as out-of-plane (**b**,**e**,**h**) and in-plane (**c**,**f**,**i**) PFM images of pulp fiber, CNF and CNF-0.3%CNT samples, respectively.

**Figure 9:**
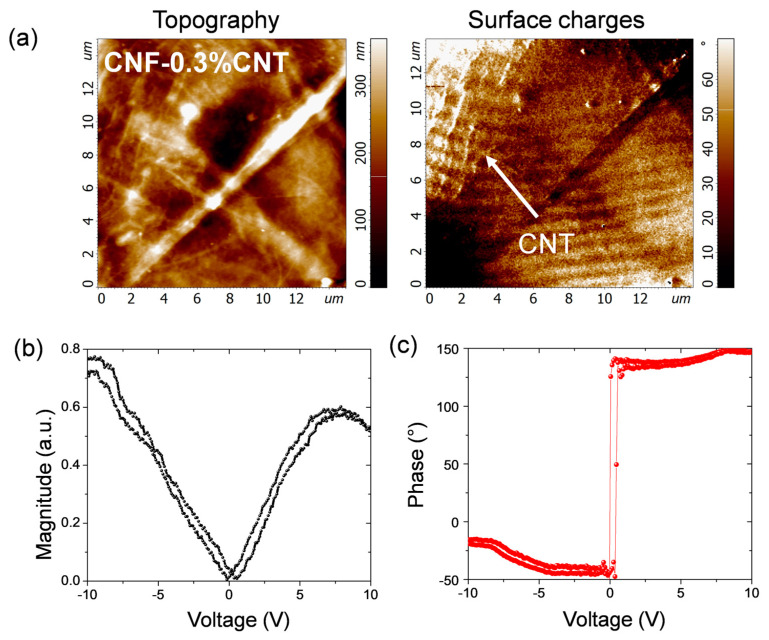
Topography and surface charge measurements obtained on the CNF-0.3%CNT sample (**a**). Force spectroscopy measurements showing the magnitude and phase of the local piezoelectric loop acquired on the CP sample (**b**,**c**).

To shed more light on the results of the macroscopic piezoelectric measurements, piezoresponse force microscopy (PFM) was employed to investigate in more depth the piezoelectric properties of pulp and cellulose nanofibers at the micro- and nanoscale levels. The morphology measurements demonstrate the fiber structure in the pulp sample, nanofiber structure in CNF and carbon nanotube fillers in the CNF-0.3CNT sample (Figure 8a,d,g). Note that the topographic image of CP was entirely taken on the surface of a single pulp fiber, which is about 10 μm wide (cf. Figure 1a) and shows a surface ribbon. The highest out-of-plane PFM response (~110 pA) related to the longitudinal d_33_ piezocoefficient was measured in the pulp sample (Figure 8b) and agrees with the higher crystallinity of this sample and the slightly higher output voltage of pure CP in tapping mode (cf. Figure S3). It may also explain the high output voltage in the cantilever mode, where besides other components (e.g., d_14_, d_15_, d_24_, d_25_, d_31_, d_32_, d_36_), d_33_ also contributes to the macroscopic piezoresponse. However, the in-plane PFM response (related to transverse d_31_) was higher in the CNF sample (Figure 8f). This could explain the macroscopical observation of the generation of higher voltage in CNF nanocomposite films than in other cellulose samples and be related to the common dipole moment of CNF nanofibers that energetically favors the in-plane direction. Hence, Figure 8d shows CNF nanofibers aligned to a common direction, and the corresponding in-plane PFM response (Figure 8f) demonstrates anisotropic behavior for these oriented structures. Moreover, the PFM results for the CNF-0.3CNT demonstrate a strong electrical screening effect of the local piezoresponse (Figure 8h,i) due to electrostatic surface charges trapped by CNT fillers (Figure 9a). The macroscopic piezoelectric measurements of the CNF-0.3CNT sample strongly depend on these trapped electrostatic surface charges revealing themselves locally in surface charge scans (Figure 9a) and macroscopically via increased current observation in the cantilever setup (Figure 7c).

Local force spectroscopy measurements demonstrate that the CNF-0.3CNT sample has very low adhesion behavior as compared to CNF and CP (Figure S4), which is related to the strong influence of electrostatic force originating from trapped surface charges. At the same time, all the samples demonstrate elastic deformation-type behavior. In order to estimate the local piezoelectric coefficients, local piezoelectric loops were measured on CP, CNF and CNF-0.3%CNT samples. Figure 9b,c display the magnitude and the phase of the loop acquired on the CP sample where a small hysteresis behavior is observed. The local piezoelectric coefficients for the d_33_ component of the piezoelectric cellulose tensor with monoclinic symmetry (space group C_2_||x_3_) were calculated to be 145, 14 and 20 pC/N for CP, CNF and CNF-0.3%CNT, respectively. It should be kept in mind that the d_33_ value for CP relates to the surface ribbon, which may be higher than the average d_33_ value for the entire pulp fiber. Nevertheless, these local d_33_ values show certain agreement with the macroscopic behavior, where pulp generates higher output voltage than CNF. As CNTs are added to CNF, the piezoresponse increases strongly in both excitation modes. However, it still remains surprising that pulp with a local d_33_ value much higher than CNF-0.3CNT (145 vs. 20 pC/N) produces a lower output voltage than the composite (in tapping mode). This points to other factors such as local fiber orientation and surface features which may increase (or reduce) the macroscopic piezoresponse.

## 4. Conclusions

The combination of three different celluloses and three types of nanocarbons was studied concerning their tribo- and piezoelectric performance in nanogenerator devices. Freestanding cellulose–nanocarbon composite films of all nine combinations were prepared by filtration methods, and the nanocarbon content was varied in each combination. Textural and microstructural characterization revealed increasing porosity of the films from 5% for CNF-based films to 35% and 52% for MCC- and pulp-based films, respectively. The nanocomposite films showed a nuanced onset of electrical conductivity depending on the cellulose type as well as on the nanocarbon filler type and concentration.

The nine combinations were tested in TENG and PENG devices, and the output voltage and current and power densities were determined as a function of the filler content. Small filler concentrations of 0.1–0.3 wt% below the onset of electrical conductivity greatly improved both the tribo- and piezoelectric voltage and power output by a factor of up to 6. The performance parameters were discussed in relation to the film properties with the aim of exposing the most important factors that influence the electrical response. The results suggest that factors such as the specific surface area of the filler, nanoscale dispersion, filler content, onset of conductivity and matrix porosity have a stronger effect on increasing the triboelectric output voltage than only the electrical conductivity of the fillers. In the case of the piezoelectric output, the cellulose crystallinity and the crystal orientation are additional important factors that influence the PENG performance.

In general, it was observed that carbon nanotubes and carbon black improve the tribo- and piezoelectric performance more than graphene nanoplatelets. Concerning the cellulose matrix, MCC and pulp are more effective in generating triboelectricity than CNF. More specifically, the highest triboelectric voltage (39 V) was obtained with MCC-0.2%CNT, while CNF-0.3%CNT rendered the highest piezoelectric voltage (0.75 V). The power densities of these materials were 60 and 0.26 mW/m^2^, respectively.

Analysis of the microscopic responses by PFM shows that pulp fibers have a higher local d_33_ (145 pC/N) than cellulose nanofibers (14 pC/N), while the macroscopic response is greatly influenced by the excitation mode (tapping vs. cantilever) and the effective orientation of the crystals relative to the mechanical stress. Adding CNTs to CNF enhances the local d_33_ (20 pC/N) and the electrostatic surface charge density, resulting in improved electrical output performance. These findings demonstrate that local piezoelectric measurements can help improve the understanding of macroscopic piezoresponses.

This work laid the foundation for more investigation that will be needed for a further substantial increase in the output performance of these paper-based energy devices. For instance, gaining control over the crystal orientation during the paper-making process could be an effective means to enhance the piezoelectric response.

## Figures and Tables

**Table 1:** Textural properties of nanocomposite films.

Sample	Thickness μm	App. Density mg/mL	Porosity %
CP-0.4CNT	72	0.69	52
MCC-2.25CNT	40	0.95	35
CNF-1.0GNP	24	1.39	5

**Table 2:** Electrical percolation threshold values (wt%) and conductivity at the percolation threshold of nanocomposite films from all nine cellulose/nanocarbon combinations.

Cellulose Matrix	Nanocarbon Filler	Percolation Threshold wt%	Conductivity ^b^ S/cm
MCC	CNTs	0.4	2.0 × 10^−5^
MCC	CB	1.0	3.1 × 10^−2^
MCC	GNPs	0.5 ^a^	-
CP	CNTs	0.3	1.8 × 10^−3^
CP	CB	1.0	1.0 × 10^−2^
CP	GNPs	0.5 ^a^	-
CNF	CNTs	2.5	2.5 × 10^−1^
CNF	CB	1 ^a^	-
CNF	GNPs	1 ^a^	-

^a^ The maximum nanocarbon filler concentration that was tested in these films which produced materials that were still nonconducting. ^b^ The conductivity at the percolation threshold.

## Data Availability

The data presented in this study are available on request from the corresponding author.

## Supplementary Materials

The following supporting information can be downloaded at: https://www.mdpi.com/article/10.3390/nano13071206/s1, Figure S1: Raman spectra of carbon allotropes; Figure S2: Electrical conductivity of MCC-CNT films; Figure S3: Tribo- and piezoelectricity voltage of all nine cellulose–nanocarbon combinations; Figure S4: Local force spectroscopy measurements of CP, CNF and CNF-0.3%CNT; Table S1: Properties of the nanocarbon fillers. Refs. [64,65,66,67,68,69,70,71,72] are cited in the supplementary materials.
