# Supplementary material for: Advanced Cellulose–Nanocarbon Composite Films for High-Performance Triboelectric and Piezoelectric Nanogenerators
Source: Nanomaterials (Basel). 2023 Mar 28;13(7):1206. doi: 10.3390/nano13071206 (PMC10097288; doi:10.3390/nano13071206)
Supplement: Supplementary file 1 [file nanomaterials-13-01206-s001.zip › nanomaterials-2285271-supplementary.pdf]

nanomaterials

*Supplementary Materials*

# Advanced Cellulose–Nanocarbon Composite Films for High-Performance Triboelectric and Piezoelectric Nanogenerators

**Jaime González [1], Ali Ghaffarinejad [2,*,†], Maxim Ivanov [3], Paula Ferreira [3], Paula M. Vilarinho [3], Ana Borrás [2], Harvey Amorín [1] and Bernd Wicklein [1,*]**

1 Materials Science Institute of Madrid (ICMM), Consejo Superior de Investigaciones Científicas (CSIC), 28049 Madrid, Spain; jaigon14@ucm.es (J.G.); hamorin@icmm.csic.es (H.A.)
2 Nanotechnology on Surfaces and Plasma Lab, Materials Science Institute of Seville (ICMS), Consejo Superior de Investigaciones Científicas (CSIC-US), 41092 Seville, Spain; anaisabel.borras@icmse.csic.es
3 CICECO—Aveiro Institute of Materials, Department of Materials and Ceramic Engineering, University of Aveiro, 3810-193 Aveiro, Portugal; ivanovmaxim@ua.pt (M.I.); pcferreira@ua.pt (P.F.); paula.vilarinho@ua.pt (P.M.V.)
* Correspondence: ali.ghaffarinejad@icmse.csic.es (A.G.); bernd@icmm.csic.es (B.W.)
† Current address: Sensors and Smart Systems Group, Institute of Engineering, Hanze University of Applied Sciences, 9747 AS Groningen, The Netherlands.

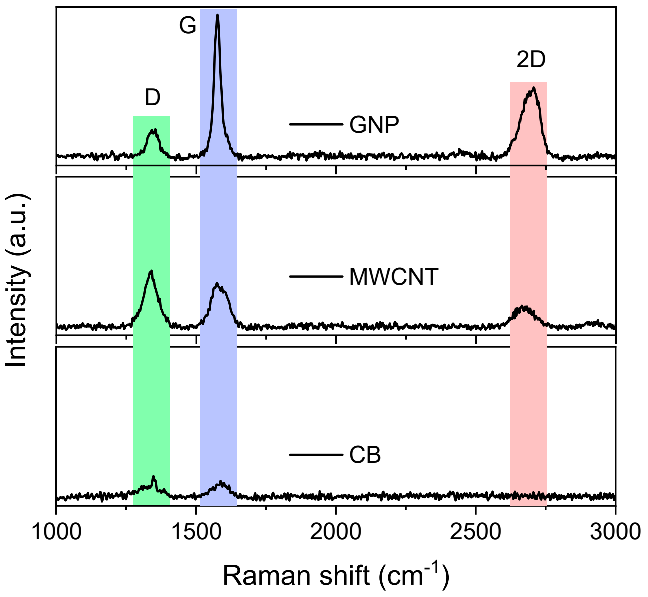


**Figure S1.** Raman spectra of CB, MWCNT and GNP. The D and G bands of $sp^3$ and $sp^2$ hybridized carbon are highlighted as well as the 2D band attributed to π stacking interactions.

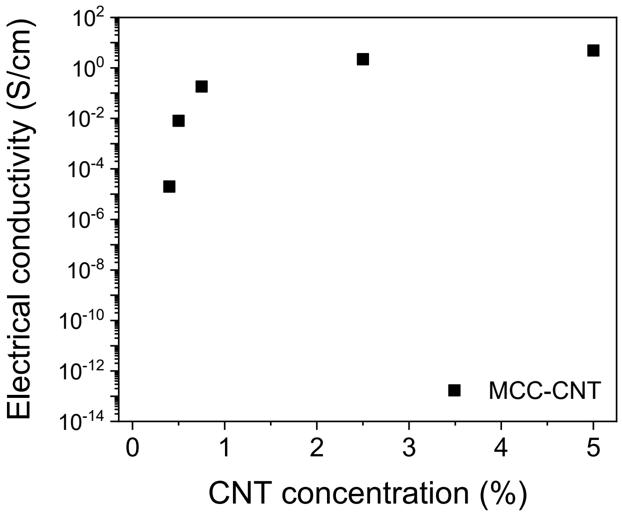


**Figure S2.** Electrical conductivity of MCC-CNT films.

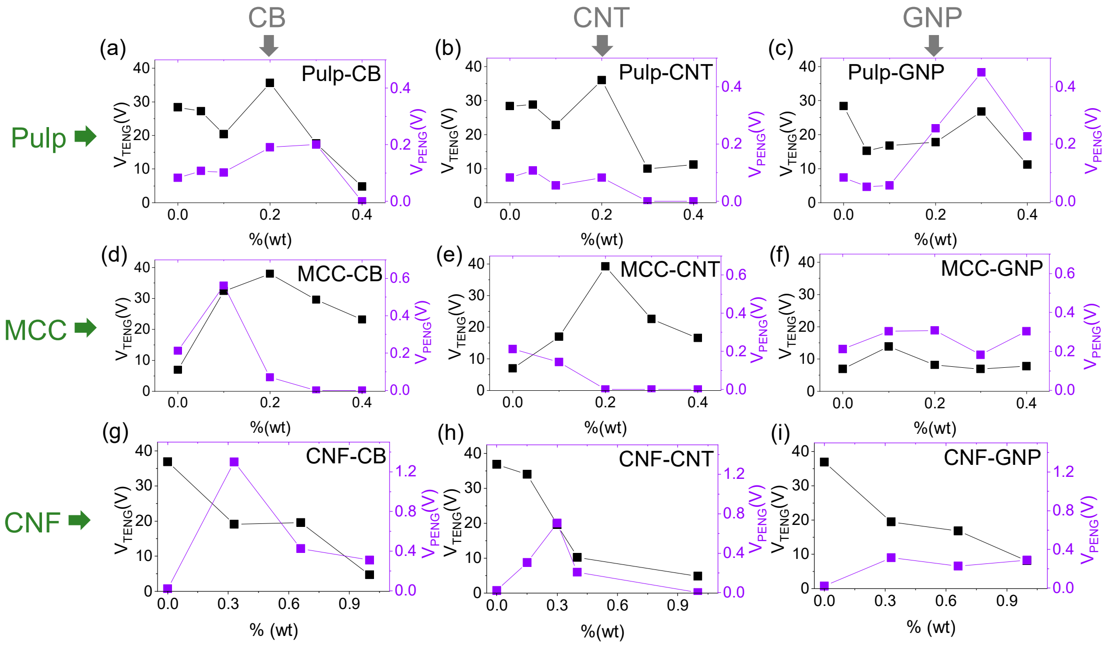


**Figure S3.** Tribo- and piezoelectricity voltage of all nine combinations as a function of the nanocarbon filler content. Pulp-CB (a), Pulp-CNT (b), Pulp-GNP (c), MCC-CB (d), MCC-CNT (e), MCC-GNP (f), CNF-CB (g), CNF-CNT (h), CNF-GNP (i).

**Table S1.** Properties of the nanocarbon fillers.

| | **Particle size (nm) Diameter x length** | **BET SSA ($m^2$/g)** | **Electrical conductivity (S/cm)** | **True density (g/$cm^3$)** | **Ref.** |
|---|---|---|---|---|---|
| CB | 35 | 250 | $10^{-4}$ | 1.80-1.98 | [1], [2],[3],[4] |
| CNT | 10 x 1,500 | 300 | $10^{1}$ – $10^{3}$ | 1.75-2.10 | [5], [6],[7],[8] |
| GNP | 5-100 x 1,000-10,000 | 30-60 | $10^{2}$ | 2.25 | [9] |

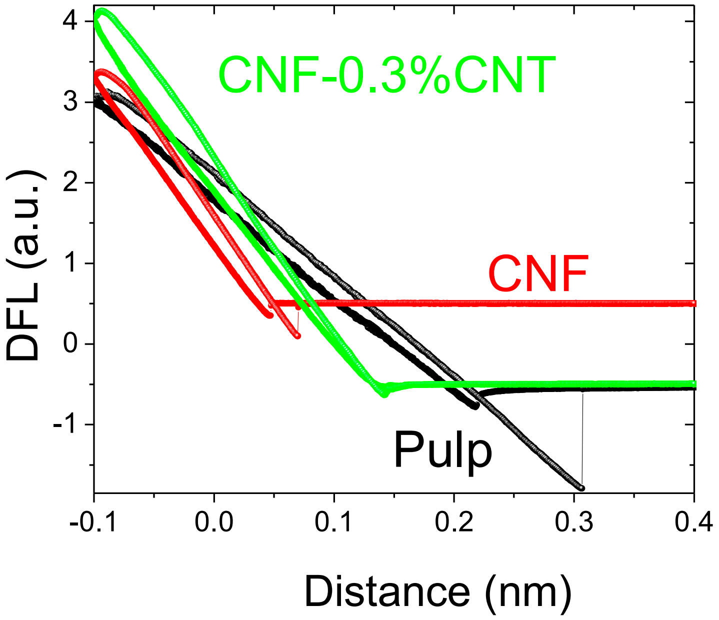


**Figure S4.** Local force spectroscopy measurements of CP, CNF and CNF-0.3%CNT samples representing laser deflection (DFL) versus tip distance.

Local force spectroscopy measurements in Figure S4 demonstrate that the CP sample is the softest one possessing higher adhesion of the sample surface, while CNF and CNF-0.3CNT are similar in rigidity (i.e. equal tracks in forward and backward behavior on DFL-distance curves) but very different in adhesion behavior (i.e. different points of tip-sample surface contact on the distance axis). The CNF-0.3CNT represents very low adhesion behavior related to the strong influence of electrostatic force due to surface charges trapped by CNT. At the same time, all the samples demonstrate elastic deformation-type behavior.

## References

1. Pantea, D.; Darmstadt, H.; Kaliaguine, S.; Roy, C. Electrical Conductivity of Conductive Carbon Blacks: Influence of Surface Chemistry and Topology. *Applied Surface Science* **2003**, *217*, 181–193, doi:10.1016/S0169-4332(03)00550-6.
2. Chen, B.-Y.; Hwang, K.-S. Comparative Study of Carbon Black and Graphite Powder as Carbon Source for PM Compacts. *Powder Metallurgy* **2010**, *53*, 51–56, doi:10.1179/174329009X409741.
3. Lu, S.; Chung, D.D.L. Viscoelastic Behavior of Carbon Black and Its Relationship with the Aggregate Size. *Carbon* **2013**, *60*, 346–355, doi:10.1016/j.carbon.2013.04.047.
4. Ehrburger-Dolle, F.; Lahaye, J.; Misono, S. Percolation in Carbon Black Powders. *Carbon* **1994**, *32*, 1363–1368, doi:10.1016/0008-6223(94)90123-6.
5. Dropsens Carbon Nanotubes - Product Information Sheet.
6. Deep, N.; Mishra, P. Fabrication and Characterization of Thermally Conductive PMMA/MWCNT Nanocomposites. *Materials Today: Proceedings* **2018**, *5*, 28328–28336, doi:10.1016/j.matpr.2018.10.117.
7. Vozniakovskii, A.A.; Kol'tsova, T.S.; Voznyakovskii, A.P.; Kumskov, A.L.; Kidalov, S.V. Powder Hybrid Nanomaterial: Detonation Nanodiamonds – Carbon Nanotubes and Its Stable Reversible Water Nanofluids. *Journal of Colloid and Interface Science* **2020**, *565*, 305–314, doi:10.1016/j.jcis.2020.01.034.
8. Kim, S.H.; Mulholland, G.W.; Zachariah, M.R. Density Measurement of Size Selected Multiwalled Carbon Nanotubes by Mobility-Mass Characterization. *Carbon* **2009**, *47*, 1297–1302, doi:10.1016/j.carbon.2009.01.011.
9. KNANO - A Graphene Company KNG-150 Graphene Nanoplatelets, Technical Product Information.
